# Feasibility of the STarT back screening tool in chiropractic clinics: a cross-sectional study of patients with low back pain

**DOI:** 10.1186/2045-709X-19-10

**Published:** 2011-04-28

**Authors:** Alice Kongsted, Else Johannesen, Charlotte Leboeuf-Yde

**Affiliations:** 1 Nordic Institute of Chiropractic and Clinical Biomechanics, University of Southern Denmark, Odense. Part of Clinical Locomotion Network, Denmark; 2 Research Department, Spine Centre of Southern Denmark, Hospital Lillebaelt Middelfart, Institute of Regional Health Research, University of Southern Denmark. Part of Clinical Locomotion Network, Denmark; 3 Master of Science in Public Health, Institute of Public Health, University of Southern Denmark, Esbjerg, Denmark

## Abstract

The STarT back screening tool (SBT) allocates low back pain (LBP) patients into three risk groups and is intended to assist clinicians in their decisions about choice of treatment. The tool consists of domains from larger questionnaires that previously have been shown to be predictive of non-recovery from LBP. This study was performed to describe the distribution of depression, fear avoidance and catastrophising in relation to the SBT risk groups. A total of 475 primary care patients were included from 19 chiropractic clinics. They completed the SBT, the Major Depression Inventory (MDI), the Fear Avoidance Beliefs Questionnaire (FABQ), and the Coping Strategies Questionnaire. Associations between the continuous scores of the psychological questionnaires and the SBT were tested by means of linear regression, and the diagnostic performance of the SBT in relation to the other questionnaires was described in terms of sensitivity, specificity and likelihood ratios.

In this cohort 59% were in the SBT low risk, 29% in the medium risk and 11% in high risk group. The SBT risk groups were positively associated with all of the psychological questionnaires. The SBT high risk group had positive likelihood ratios for having a risk profile on the psychological scales ranging from 3.8 (95% CI 2.3 - 6.3) for the MDI to 7.6 (95% CI 4.9 - 11.7) for the FABQ. The SBT questionnaire was feasible to use in chiropractic practice and risk groups were related to the presence of well-established psychological prognostic factors. If the tool proves to predict prognosis in future studies, it would be a relevant alternative in clinical practice to other more comprehensive questionnaires.

## Background

Low back pain (LBP) is common and most cases of LBP are handled either without any contact to the health care system or in primary care [1,2]. However, some LBP patients develop severe or long-lasting pain with far-reaching consequences both personally and socioeconomically [3]. It is widely accepted that patients at risk of lasting back disability should be identified early in their course of LBP in order to prevent chronicity, and much effort has been put into investigating factors that predict non-recovery in LBP [4,5]. Although variability in research methods and quality limits what can be concluded about useful predictors in LBP, it seems that self-reported information on symptoms and beliefs about LBP is as valuable from a prognostic angle as data from the clinical examination [4,5]. It is therefore worthwhile to construct questionnaires focusing on such established prognostic indicators that can easily be filled in directly by patients.

The STarT back screening tool (SBT) was introduced as a tool that can assist general practitioners' decision- making concerning initial treatment options in primary care [6]. It consists of nine questions covering aspects of fear avoidance beliefs, depression, disability and presence of leg pain and neck/shoulder pain. Patients are allocated into one of three subgroups (low, medium or high risk of chronicity) based on the obtained score. The authors suggest that the low risk group only needs a 'light' intervention with e.g. analgesics and advice, the medium group requires treatments involving elements such as exercises or manual therapy, and that a combination of physical and cognitive-behavioral approaches should be considered for the high risk group [6].

The SBT has been validated against well-established questionnaires regarding disability and psychological parameters and its psychometric performance was shown to be similar to that of the longer Orebro Musculoskeletal Pain Screening Questionnaire [7]. So far, the tool has not been tested outside the United Kingdom and it has not been validated in relation to identifying patients with different treatment needs.

The aims of this study were 1) to test whether chiropractic patients in Denmark were able and willing to fill in a Danish version of the SBT, 2) to find out whether this tool is able to identify the three subgroups also in this cohort, and 3) to examine whether patients in the three expected subgroups differ regarding gender, age, symptoms, depression, fear avoidance beliefs, and catastrophising coping strategies.

## Methods

A cross-sectional study was conducted with data collection in 19 chiropractic clinics that constitute the practice-research unit organized under the Nordic Institute of Chiropractic and Clinical Biomechanics. These clinics are geographically scattered throughout Denmark and have volunteered to participate in data collection upon invitation. In Denmark there is no requirement of a referral for chiropractic care and most patients see a chiropractor on their own initiative. The state pays some of the fee through a collective agreement with the chiropractic profession, but most (approximately 80%) is paid by the patient. The project was presented for the local ethical committee who stated that it did not need approval.

### Procedures

The objectives of the project as well as practical procedures were described to the participating chiropractors at a meeting. Questionnaires and project procedures in writing were handed over at the meeting. On the first day of the data-collection the clinicians or their secretaries were contacted by phone to clarify any questions about the procedures. Another call was made at the end of the first week of data-collection to make sure they were all well underway, and this was repeated in weeks two and three, if considered necessary.

Patients with LBP were invited to participate when presenting to the clinics, and those who agreed to fill in a questionnaire were asked to do so in the waiting room. Afterwards, the completed questionnaire was put into a sealed envelope and returned to the chiropractor or secretary. Once a week the questionnaires were sent to the research unit. In a few cases the questionnaires were filled in by patients at home and afterwards returned to the clinic.

### Patients

All patients between 18 and 67 years of age, who were consulting the chiropractor because of a LBP problem and were able to read and speak Danish, were qualified for participation.

### Questionnaires

**Figure 1:**
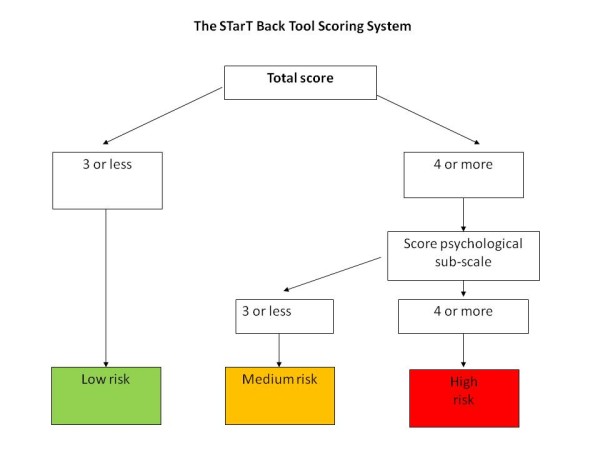
**Allocation of patients into risk groups according to their SBT-scores**. The illustrating was adapted from http://www.keele.ac.uk/research/pchs/pcmrc/dissemination/tools/startback/.

The questionnaire package consisted of the STarT back screening tool (SBT)[6], the Major Depression Inventory (MDI) [8], the Fear Avoidance Beliefs Questionnaire (FABQ) [9], and the Coping Strategies Questionnaire (CSQ) [10]. The allocation of patients into the three SBT risk groups is described in Figure 1. The purpose and scoring of the questionnaires are summarized in Table 1. The dichotomizing of the MDI and the FABQ were based on previous recommendations [8,9]. The CSQ consists of six subscales, each identifying the use of one coping strategy. For the present study we used only the subscale related to catastrophising. There is no consensus about a cut-point on the catastrophising sub-scale of the CSQ, therefore, based on the distribution in our study, we decided to consider scores of 16 or more to indicate a high use of catastrophising strategies, as this was considered the point for the split between the majority of observations and the right hand tail of the estimates.

**Table 1:** Overview of the questionnaires used in the study

Questionnaire	Intended to evaluate	Possible range of scores	Sub-divisions used
SBT	Risk of chronicity	0 - 9 (overall)	Low risk: Overall < 4
		0 - 5 (psychological sub score)	Medium risk: Overall ≥ 4 and psych. sub scale < 4
			High Risk: Psych. sub scale ≥ 4

MDI	Depression	0 - 50	> 24: depressed

FABQ	Fear Avoidance Beliefs	0 - 66	> 48: high fear avoidance

CSQ	Use of six coping strategies. Catastrophising was the only domain used for the present study	Catastrophising: 0 - 36	≥ 16: high use of catastrophising

SBT: STarT back screening tool. MDI: Major depression inventory.

FABQ: Fear avoidance beliefs questionnaire. CSQ: Coping strategies questionnaire.

In addition to the above described questionnaires, patients were asked about demographic factors, pain duration of the present episode (0 - 2 weeks, 2 weeks - 3 months, > 3 months), total number of pain days the preceding 12 months (< 30 days, ≥ 30 days), and number of days with LBP during the previous two weeks (0 - 14).

### Data Analysis

#### The SBT questionnaire

Since the SBT holds only nine items that represent different domains, strict criteria for completion were defined. If an item was missing from the SBT, the missing value was replaced with the most frequent answer (0 or 1) to the remaining questions. If more than one item were missing, the entire scale was excluded.

User friendliness was established by noting the number and nature of unanswered questions. Whether the three SBT subgroups existed in the cohort was examined by noting the proportions of patients allocated into these groups.

#### The other questionnaires

Missing scores on the MDI and the FABQ scales were replaced by the average of the completed ones, if no more than two items were missing. Since the catastrophising domain of the CSQ only consisted of 6 questions, we used the average value on that scale to impute the missing value, if only one item was missing. In case of more missing values the scales were excluded. Imputation was performed in four, twenty-five and four scales of the MDI, FABQ, and CSQ respectively.

#### Associations between the SBT and the other questionnaires

Associations between the SBT risk groups and the MDI, FABQ, and CSQ were tested by means of linear regression with robust variance estimation using the SBT risk groups as a categorical variable. Prior to the regression analyses it was tested whether age and gender were equally distributed in the SBT groups. Since this was the case, the analyses were conducted without any adjusting. The dichotomised MDI, FABQ, and CSQ scores were used to calculate the sensitivity, specificity and likelihood ratios in relation to the SBT's diagnostic performance. Furthermore, prior and posterior probabilities of high scores on these scales were calculated to evaluate how knowledge of the SBT categories would alter a patient's "risk profile".

Data were double entered into Epidata (The EpiData Association, Odense, Denmark), corrected when necessary and transmitted to STATA 10.1 (StataCorp, Texas, USA) for the analyses.

## Results

### Study Population

A total of 607 questionnaires were distributed to the chiropractic clinics, and 543 questionnaires were completed between the 23^rd ^of November and the 15^th ^of December 2009. Twenty-four of these questionnaires were discarded because the respondents were not within the age-limits defined in the inclusion criteria, and the participants hence consisted of 519 subjects (255 females, 207 males, 57 did not report gender). The involved clinics recruited from seven to forty patients each (median = 31, IQR 29 - 35). The mean age was 43 years (67 subjects did not report age).

The participating patients were almost equally distributed among acute (30%), sub acute (36%), and chronic LBP (34%). Fifty-seven percent reported to have had pain for more than 30 days during the preceding year, and during the past two weeks a median of 8 days with LBP was reported (Interquartile range 4 - 14). Daily pain during the last two weeks was reported by 31% of the participants, and 43% reported that pain has spread to the leg(s) within the preceding two weeks.

### Completion of the SBT

A total of 451/519 (87%) of the SBT questionnaires were complete. One item was missing in 24 questionnaires, and another 44 patients, who were hence excluded, had missed more than one item. Hence, the SBT was adequately completed by 475/519 (92%) of the patients; 244 females, 194 males, 37 sex not reported. The mean age in this final study sample was 43 (range 18 - 67).

When only one item was missing, it was most frequently the answer to 'Worrying thoughts have been going through my mind a lot of the time in the last 2 weeks' (5 cases) or to 'In general in the last 2 weeks, I have not enjoyed all the things I used to enjoy' (5 cases).

### Distribution of patients on the SBT groups

The overall SBT scores ranged from 0 to 9 (median 3, IQR 1 - 5). The proportions of patients in the three risk groups were 59% (95% CI 55 - 64%) in low risk, 29% (95% CI 25 - 33%) in medium risk, and 11% (95% CI 8 - 14%) in high risk. The proportion of low risk patients varied between the data collecting clinics from 52% to 73%, and the proportion of high risk patients from 4% to 33%.

### Patient characteristics in the SBT groups

#### Gender, age and symptom characteristics

Associations between the risk groups and the baseline characteristics appear from Table 2. The high risk patients had more days with LBP both the preceding year and the preceding two weeks, and tended to have had a longer duration of the present episode. Regarding duration the low and medium risk groups were very similar, whereas number of LBP days during the past two weeks increased for each SBT risk level. Fewer patients in the low risk group reported leg pain as compared to the other groups, which is partly explained by leg pain being one of the SBT items. Gender and age were equally distributed over the risk groups.

**Table 2:** Distribution of findings in 475 chiropractic patients in relation to the three STarT back screening tool risk groups.

	Low Risk n = 282	Medium Risk n = 139	High Risk n = 54	p-value
Duration, %				0.2
< 2 weeks	32	34	17	
- 3 months	34	36	44	
> 3 months	34	30	39	

> 30 days LBP preceding year, %	51	59	76	< .01

Leg pain, %	26	64	69	< .01

Number of LBP days during the last 2 weeks, median (IQR)	7 (3 - 12)	10 (6 - 14)	14 (10 - 14)	< .001

IQR = Interquartile range.

#### Associations between the STarT Back Screening Tool and the Major Depression Inventory

**Figure 2:**
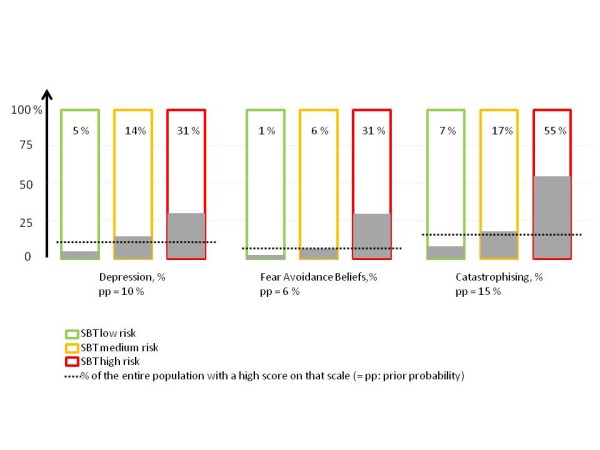
**The proportion of patients with high scores on depression, fear-avoidance or catastrophising within the three SBT risk groups in 475 chiropractic patients**.

The MDI was adequately completed by 471 patients. The scores ranged from 0 to 42 (median 9), and 10% had a score of > 24 indicating the possibility of at least a moderate depression. The continuous MDI scores were positively associated with the SBT risk groups showing a dose-response relation (Table 3). The proportions of patients with signs of depression increased from 5% in the low risk group to 31% in the high risk SBT group (Figure 2). The low risk group had a lower post-test risk of being depressed than the prior probability, whereas the posterior risk of the medium risk group did not differ from the risk of the entire population (Table 4). The high risk group had a high specificity for depression and an almost four times increased likelihood of depression, but the sensitivity was only 33% (Table 4).

**Table 3:** Associations between continuous scores on psychological questionnaires and three SBT risk groups

	Regression coefficients (95% CI)	
**MDI**		p < 0.001
Low risk*	0	
Medium Risk	6.6 (5.1 - 8.2)	
High Risk	12.2 (9.5 - 15.0)	

**FABQ**		p < 0.001
Low risk*	0	
Medium Risk	5.7 (3.1 - 8.3)	
High Risk	17.7 (13.0 - 22.3)	

**Catastrophising**		p < 0.001
Low risk*	0	
Medium Risk	3.8 (2.5 - 5.1)	
High Risk	9.8 (8.0 - 11.6)	

* Reference category. CI: Confidence interval. MDI: Major depression inventory. FABQ: Fear avoidance beliefs questionnaire.

**Table 4:** Llikelihood ratios, sensitivities, and specificities for the three SBT groups' diagnostic performance in relation to identifying patients with high scores on three more comprehensive questionnaires

	Pretest risk of high scores, % (95% CI)	Neg. LHR (95% CI)	Pos. LHR (95% CI)	Sensitivity % (95% CI)	Specificity % (95% CI)
**MDI**	10 (8 - 14)				
Low risk		2.01 (1.63 - 2.48)	.42 (.26 - .67)	27 (15 - 41)	37 (32 - 41)
Medium Risk		.82 (.65 - 1.04)	1.46 (1.01 - 2.11)	41 (27 - 56)	72 (68 - 76)
High Risk		.74 (.61 - .90)	3.83 (2.30 - 6.37)	33 (20 - 48)	92 (88 - 94)

**FABQ**	6 (4 - 8)				
Low risk		2.35 (1.96 - 2.81)	.18 (.06 - .52)	11 (2 - 29)	38 (33 - 43)
Medium Risk		1.00 (.78 -	1.00 (.55 - 1.82)	30 (14 - 52)	70 (66 - 75)
High Risk		1.29) .44 (.28 - .70)	7.21 (4.63 - 11.2)	59 (39 - 78)	92 (89 - 94)

**Catastrophising**	15 (12 - 19)				
Low risk		2.10 (1.72 - 2.55)	.41 (.28 - .61)	27 (17 - 39)	35 (30 - 40)
Medium Risk		.95 (.80 - 1.13)	1.12 (.78 - 1.63)	32 (22 - 45)	71 (66 - 76)
High Risk		.63 (.52 - .77)	6.67 (4.14 - 10.8)	41 (29 - 53)	94 (91 - 96)

LHR: Likelihood ratio

#### Associations between the STarT Back Screening Tool and the Fear Avoidance Beliefs Questionnaire

The FABQ scores, from 465 patients who completed the scale, ranged from 0 to 66 (median 22) and 6% had high fear avoidance beliefs. This proportion ranged from 1% - 31% in the SBT risk groups (Figure 2), and fear avoidance scores were positively associated with risk group (Table 3). As for depression, the high risk group had a high diagnostic performance, the low risk group increased the likelihood of non-fear avoidance, and the medium group had a profile similar to that of the total population (Table 4).

#### Associations between the STarT Screening Back Tool and catastrophising

The catastrophising sub-scale of the CSQ was available from 463 of the patients. Scores were 0 - 36 (median 8), with 15% categorized as high on catastrophising. Catastrophising was positively associated with risk group (Table 3), and the proportions with high scores ranged from 7% in the low risk group to 55% in the high risk SBT group (Figure 2). The diagnostic properties of the SBT groups in relation to catastrophising resembled what was seen for fear-avoidance (Table 4).

#### Co-existing psychological factors

**Figure 3:**
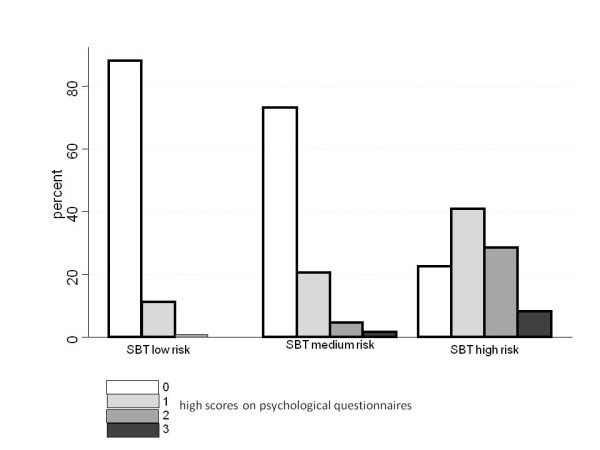
**Proportions of patients within the three SBT risk groups with high scores on 0, 1, 2, and 3 psychological questionnaires**.

Data were available from all questionnaires in 453 patients. Among these, 76% had none of the three measured psychological factors, 17% had one, 5% two and 2% (7 patients) had high scores on all three scales. In the low, medium and high risk SBT groups 12%, 28%, and 76%, respectively, had high scores on at least one of the other scales. The number of positive scores on psychological factors increased from the low risk to the medium and high risk groups (Figure 3).

## Discussion

This study tested the 9-item SBT and compared it to three large and well-known psychological questionnaires in a cohort of Danish primary care patients. The SBT is appealing to primary care clinicians since most patients would be able to complete it in short time and it can be scored easily on the spot by the clinician. The studied cohort was able to complete the SBT with very few missing values, and patients were distributed on the three pre-defined risk groups. The proportion of patients with a "risk profile" was rather low both on the SBT and regarding depression, fear- avoidance beliefs, and catastrophising from 6% with high scores on fear avoidance beliefs to 15% expressing a catastrophising coping strategy. Still, close to 25% of the study population had high scores on at least one of the psychological questionnaires. If the investigated factors are important prognostic factors in this population, it would be highly relevant to identify such patients. This remains to be clarified.

### Distribution of patients on SBT subgroups

Compared to the population used for the initial validation of the SBT [6], a larger proportion of our population was in the low risk group (59% versus 47%). This difference may be a result of the two populations being recruited from different settings, general vs. chiropractic practice, or of one being from the UK and the other from Denmark. There is presently an ongoing validation of the SBT-cut points in Danish primary care that will reveal whether the scale should be interpret differently in these patients (Morsø, L. Personal communication).

Since almost 60% of the present population was in the low risk group, there is potentially a high number of patients seeking chiropractic care, who need only advice and "minimal care". If validation studies support the use of the established cut-points in Danish patients, and if randomised trials confirm the hypothesis that the low risk group only needs minimal care, this could be important for allocation of resources within back care.

### Does the SBT identify patients with a psychological risk profile?

We tested the SBT against three comprehensive questionnaires regarding psychological prognostic factors and found significant associations between the SBT risk groups and scores on the other scales. The presence of high scores on depression, fear avoidance beliefs, or catastrophising increased significantly from the SBT low risk group, over the medium group to the high risk group. A patient in the SBT high risk group had a 10-fold increased likelihood of having a high score on at least one of the three psychological questionnaires as compared to the probability prior to knowing the SBT risk group. Both the low and the high risk group had useful diagnostic properties as compared to the chosen questionnaires. *Not *being in the low risk group increased the likelihood of psychological risk factors to some extent and *being *in the high risk group was related to a marked increased likelihood of a risk profile.

These results support the assumption that the SBT can assist clinicians' detection of patients for whom a psychological assessment is likely to be relevant. The questionnaires that we used to measure depression, fear avoidance beliefs and catastrophising differed from those used in the original validation of the SBT, and the present results adds to the impression that the SBT actually distinguishes between patients with truly different profiles.

### Do the SBT groups differ in relation to symptom characteristics?

The intention of the SBT is not to detect those patients who have a certain psychological profile, but rather to identify the patients, who have the most severe back pain complaints, and who, therefore, possibly have a special need for care. The present study did not compare SBT groups to pain severity or disability scores, but we found that patients in the high risk group more often reported a high number of LBP days the preceding year, that the present episode had more often lasted for more than two weeks, and that they were more likely to have had daily pain during the preceding two weeks as compared to patients in the low and medium risk groups. A higher number of LBP days during the previous year was earlier shown to be a negative prognostic factor in primary care LBP patients [11], and the larger proportion of patients with > 30 LBP days the preceding year in the high risk group as compared to the other groups supports the notion that the predictive value of the risk groups is worth investigating.

### Strengths and limitations of the study

The study was carried out in a large population recruited from nineteen clinics and we believe that this cohort represents Danish chiropractic patients well. The questionnaires were well completed and only a very small number was not included in the analyses due to missing values. We only tested the SBT in relation to the psychological domains and did not include pain intensity or disability scales. Therefore our study is limited to describe the distribution of a few known risk factors, which are not very prevalent in the present study population. Ongoing work will describe the relationship between other health domains and the SBT risk groups in Danish chiropractic patients.

It should be noted that there is no documentation for the chosen catastrophising cut-point, and the proportion found to be catastrophisers should be interpret with caution. If we had instead considered patients with an above-median score to be catastrophisers this definition would obviously include a much higher number of patients.

### Clinical relevance

Results on the predictive value of the SBT have to our knowledge only been published from one study so far [6]. In UK general practitioner patients, the SBT risk groups did relate to risk of non-recovery after 6 months. If the domains included in the SBT are modifiable, as they instinctively would be, this is a promising tool to guide the intervention offered to patients. The potential gain from offering targeted treatment as guided by the SBT is presently studied by Foster et al. [12]

It is still to be tested whether the SBT is useful as a predictor of prognosis in other populations and on the mere basis of cross-sectional studies such as this one it is not possible to recommend whether clinicians should use the SBT. What we can conclude based on the present study is that the SBT is feasible to use in chiropractic clinics, and with the low prevalence of psychological risk factors in this population, it is most relevant to screen for these risk factors with a short and general tool instead of having to use three large questionnaires that would be irrelevant to most patients.

### Future research needs

There is a need for testing the prognostic value of the SBT in different patient populations. Thereafter RCTs, specifically designed for testing if treatment effects differ in the SBT subgroups, should be conducted to evaluate if interventions can be delivered more efficiently when clinical decisions are guided by the SBT risk groups. This study was a small step towards testing a tool that may in coming studies show helpful when deciding which patients we should spend the most resources on.

## Competing interests

The authors declare that they have no competing interests.

## Authors' contributions

All the authors participated in planning of the study. EJ coordinated the data collection. AK was responsible for the design, did the data analyses, and drafted the manuscript. EJ and CLY have critically revised the manuscript and all authors have read and approved the final manuscript.
